# Wearable Smart Bandage-Based Bio-Sensors

**DOI:** 10.3390/bios13040462

**Published:** 2023-04-06

**Authors:** Arie Levin, Shu Gong, Wenlong Cheng

**Affiliations:** Department of Chemical & Biological Engineering, Faculty of Engineering, Monash University, Clayton, VIC 3168, Australia

**Keywords:** flexible wearable sensors, smart bandages, wearable electronics, biosensors, regulatory, wound dressing

## Abstract

Bandage is a well-established industry, whereas wearable electronics is an emerging industry. This review presents the bandage as the base of wearable bioelectronics. It begins with introducing a detailed background to bandages and the development of bandage-based smart sensors, which is followed by a sequential discussion of the technical characteristics of the existing bandages, a more practical methodology for future applications, and manufacturing processes of bandage-based wearable biosensors. The review then elaborates on the advantages of basing the next generation of wearables, such as acceptance by the customers and system approvals, and disposal.

## 1. Introduction

**Figure 1:**
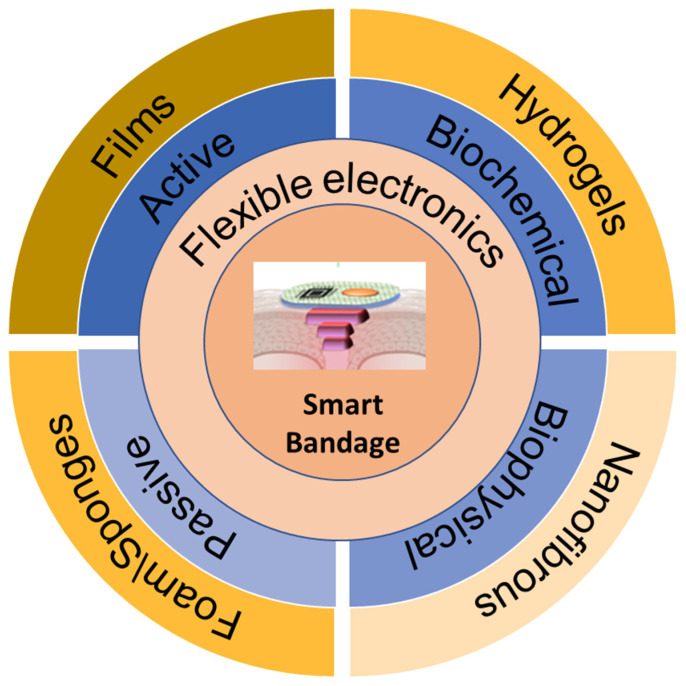
Schematic illustration of the smart bandage structure levels.

Despite advancements in the field of flexible electronics and material science, wearable biosensors have become only partially available to the public. Wearable electronic devices were brought to the awareness of the general public at the time of their first appearance in the late 1970s [1]. Initially, their goal was to facilitate the use of existing electronic devices; however, with advancements in electronics, their evolution accelerated. Today, wearable devices have adopted the form of gadgets that incorporate electronics to simplify and ameliorate daily activities, and to perform continuous data collection about users’ routine activities. They have enabled the real-time monitoring of vital signs, and provide monitoring, diagnostics, and performance analysis. Advances in materials and the design of soft electronics, skin-like sensors and conformal wearable electronic devices are drawing significant attention, but are still far from being commonly used in real-world applications due to the challenges such as usability, reliability, and durability. While soft and flexible substrates such as textiles and polymers are being explored, bandages have attracted some attention due to their ability to adhere to the skin and provide conformal contact. Knowledge of electronics’ application to human skin, mass manufacturing methodology, and the integration of sensors into the mainstream consumer market [2] has not been fully implemented. On the other hand, much research, development, and funds are being deployed in the field of acute and chronic wound treatments, as it has an enormous and increasing impact on healthcare systems worldwide [3]. It is considered a severe growing burden on the global healthcare system, and as a result, a plethora of different types of bandages have been introduced into the market in the last several decades. Many resources can be leveraged to bring advancement in the field of wearables to the market, such as the availability of a large variety of bandages, the vast amount of knowledge being accumulated in the healthcare system, and a global market size expected to pass USD 28 billion by 2029 [4], which can be viewed as a driving force towards faster adaptation of the bandages in development. This article aims to present the bandage as the basis of wearable technology by introducing a detailed background to bandages and the development of bandage-based smart sensors. In addition, it will address the big gap between the research outcomes of advanced bandages and the available products on the market. It does so by presenting an-up-to date overview of the wearables and biosensors fields and constructing a clear picture of the availability, usability, and technical characteristics of existing bandages (Figure 1). The use of bandages can minimize the environmental footprint of modern electronics and contribute to environmental sustainability. Research into waste disposal during the pandemic, waste management in general [5,6], and the recycling of bandages for use in other industries [7] can benefit from acceptance of bandage-based wearables. In addition, the use of bandages in the development of the next-generation wearables will help shorten the path to market via mainstream acceptance and overcoming regulatory obstacles. Moreover, a more practical development and production-oriented methodology could be considered as the basis of future applications and manufacturing processes of bandage-based wearable biosensors.

## 2. The Evolution of Bandages

**Figure 2:**
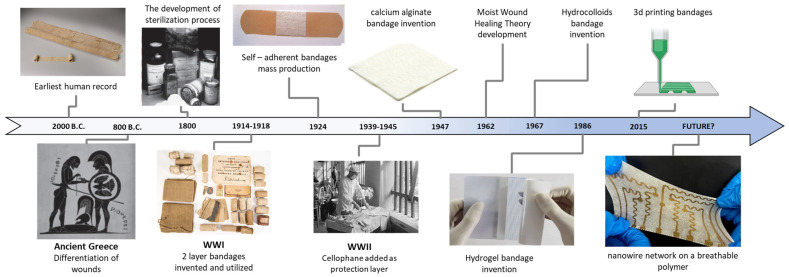
The development of bandages through time and major milestones throughout the last five millennia. Most of the advancements were achieved in the 20th century, particularly during the wars of the era. The adherence to skin tissue was the catalyst of using a two-layer structure [12,13,14], and the industrial revolution introduced mass production to the field. Earliest human record [15], ancient Greece [16], sterilization [17], WWI image [18], WWII image [19] Reproduced under the CC_BY license, Hydrogel Reprinted with permission from Heliyon, Copyright 2020, Elsevier [20], 3d printing Reprinted from “3D printing nozzle (multilayered)”, by BioRender.com (2023) Retrieved from https://app.biorender.com/biorender-templates, accessed on 9 March 2023. Future Reprinted with permission from Materials Today, Copyright 2022, Elsevier [21].

The usage and design of the bandage as we know it today is a five millennia-long journey [8,9] (Figure 2). From Ancient Egypt, where linen (for the covering of wounds and exudate removal) and disinfectants such as wine, vinegar, and honey were used, to the Ancient Greeks, who presented us with the first written description of different types of wounds. The Homeric poems, written in 800 B.C., are considered the highest intellectual product of the era, and included descriptions of daily life during the siege of Troy (the Iliad). The poems described more than 130 different types of wounds and injuries that were inflicted upon soldiers during the battle of Troy, between the 12th and 13th centuries B.C [10,11,12]. Even with such a long period of existence, big advancements in the field were not achieved up until the 19th century, as the progression during the Dark Ages and Renaissance eras focused on perfecting the methodologies of existing solutions (animal- and plant-based wound treatments).

The importance of sterilization [12,22] was the biggest advancement in the 19th century, and was introduced by Johnson & Johnson’s educational manual, *Modern Methods of Antiseptic Wound Treatment*; it helped to explain how to prevent the growth of infection-causing microorganisms during surgery [23]. The major advancements that helped bandages to evolve into those commonly used today took place both in the geopolitical and academic arenas. The major wars of the 20th century [18,24,25,26], (WWI, WWII, and the Vietnam War) made first-aid kits and the bandages in them an essential part of the treatment of wounded soldiers during combat. Such was the work of Bloom [27], with sterilized cellophane on prisoners with burn injuries during WWII, and Bull et al., who developed a transparent film dressing made of nylon [28].

Other milestone achievements, such as “Moist Wound Healing Theory” by Prof. George D. Winter in 1962 [29], the 1968 study by Z.T Piskozub, “Removing Excess Exudate” [30], and the 1982 J.C Lawrence study, “Importance of Adherence to Tissue Damage” [31], helped to shape today’s more advanced bandages, as they redefined their main purpose. Moreover, the use of plastic materials such as Polyethylene (PE), Polyurethane (PU) and nylon for wound dressing marked the introduction of advanced materials to the bandage field. At the beginning of the 21st century, advances in 3D printing and flexible electronics [32,33] offered new opportunities in the design of future smart bandages. Such an active bandage may revolutionize the medical fields of chronic wounds and personalize medical treatments to improve health outcomes [34,35,36].

## 3. Bandage Design

**Figure 3:**
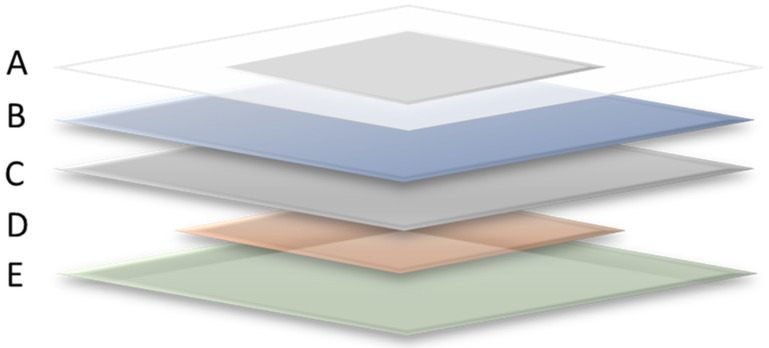
General design of the majority of bandages, A: Delivery Method. A paper-based skeleton for improving the on-skin application (product- and company-dependent). B: Baking. Acts as the spine of the bandage with additional roles such as that of a protective barrier against fluids, microorganisms and other impurities; it keeps the wound under moisture balance for optimal healing. C: Adhesive film. Enables gentle conformability to the skin; silicon or acrylic adhesives are commonly used. Important for keeping the dressing in place. D: Absorbent layer. Absorbs the excess of fluids, exudate, and impurities from the wound surface. E: Release liner. Low-friction film, released before application. Bandages may come in many forms such as foam, nonwoven materials, absorbent fibers and more. The main structure is the combination of layers B, C and D.

The design of today’s bandages was established on the basis of the four stages of wound healing (hemostasis, anti-inflammation, proliferation and remodeling) [37,38] and a given patient’s characteristics (depth of wound, surfaces of wound, skin types, exudates and moisture level) [39,40,41,42,43]. These stages overlap and occur in a specific and connected order [44,45]. The length of the healing progress and the advancements of these stages are affected by many parameters, including wound type [46], the physiology of the patient, and the surrounding environment the patient resides in. As a result, each stage of healing has specific requirements of the bandage that is being used. With that said, the process of classification of the wound based on the type and origin does exist, but an all-purpose ideal dressing does not. This lack of a universal solution raises the need for a different type of bandage that is tailored to the specific patient’s background and the general environment they reside in. Hence, the selection of the material and design of the bandage for a particular wound is important to achieve a faster and complete healing process. In general, the layout of any bandage may be summarized by the number of layers in it (Figure 3). Layer A is typically made of paper or plastic, and its functions include keeping the bandage’s form up to the application on the patient. Layer B is typically made of polypropylene or polyethylene terephthalate (PET), and is considered the bandage’s spine, as it dictates the bandage’s mechanical characteristics and desired performance. Layer C is the adhesive layer that allows the bandage to bond to the surface, where the type and thickness of the adhesive determines the strength of the bond. Silicon-based and acrylate adhesives are often used. Layer D consists of some type of absorbent material such as cloth or foam. Layer E is the liner that provides a clean, consistent surface on which to coat the adhesive, and protect the adhesive surface from exposure and damage. It is mostly made out of poly-olefin-coated paper, PET, or polypropylene [47]. Based on these principles and characteristics, a vast range of bandages have been designed in recent decades to improve the treatment of chronic ulcers and wounds, which vary by size, material, design and application.

**Figure 4:**
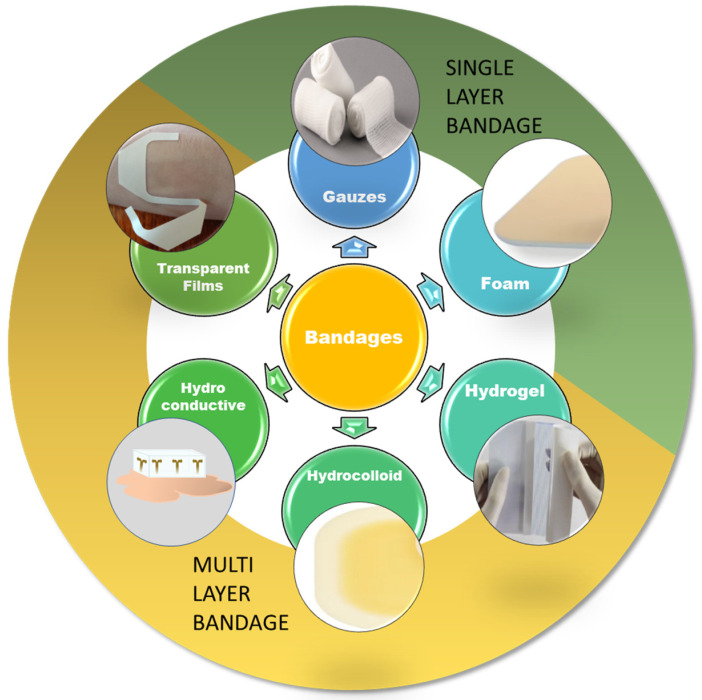
The traditional bandages and their grouping into single- and multiple-layer structures. Hydrogel reprinted with permission from Heliyon, Copyright 2020, Elsevier [20].

Conventional bandages can be divided into single or multilayer designs and categorized into six different types, as shown in Figure 4: gauzes, foam, hydrogel, hydrocolloid, hydro-conductive and transparent nanofibrous films.

Gauzes are the cheapest, most commonly used and highly absorbent single-layer bandage available today. The name gauze is often used to describe a wide range of products that is then divided into two main categories: woven and non-woven. The woven products are composed of 100% cotton yarns, and have been manufactured by the same method for centuries. They are more absorbent, but more traumatic to remove and have the tendency to shed fibers. The non-woven products were introduced in the last century, and are generally made of rayon or other synthetic fiber blends that address the disadvantages of woven gauzes [48,49,50]. As the oldest known bandages on the market, gauzes have many advantages in being a good platform for mass-produced biosensors, such as their ability to absorb a large amount of exudate and body fluids, their easy adaptation to the human skin and their economic advantage. However, gauzes suffer from many limitations (Table 1), including a lack of protection from external impurities and traumatic removal.

An additional single-layer bandage design may be achieved with foam. Foam-based bandages, similar to gauzes, can absorb large amounts of fluid while enabling gas exchange and good thermal management, possibly due to their porous structure, which enables liquid to flow into air-filled cavities by capillary action. The most commonly used material is polyurethane, but silicone-based products are used as well, mainly as an adhesive wound contact layer [14,56]. The big advantage of foam bandages vs. gauzes is they do not leave residues and microfibers; however, they do not have the ability to adhere to the surface. Unlike gauze, which can be wrapped around the location of the wound or the monitored area, foam bandages need an adhesive layer to stay in place and an additional protective layer from external impurities (Table 1).

Hydro-conductive bandages are the latest class of bandages that were introduced with a specific multi-layer structure. Drawtex is an example of such a product; it consists of three layers that use a combination of hydrostatic, electrostatic and capillary actions to perform hydro-conductive debridement. Both the inner and outer absorbent layers are made of polyester fibers, while the middle screen layer consists of 20% cotton and 80% polyester. The advantage of a hydro-conductive bandage is its ability to absorb liquid (which is similar to that of foam bandages) while retaining its integrity [57,58,59]. Its excellent exudate absorption, debris, bacteria and impurity clearing [60,61] and the non-traumatic removal of the bandage mean it is a step closer to the ideal bandage characterization. Nonetheless, the high frequency of replacement needed, and its specific use (burn wounds) make it an adequate solution for chemical biosensors with very specific objectives, high costs and additional layers to be considered in the manufacturing stages.

Hydrocolloids are typically made by a combination of gel-forming agents (carboxymethylcellulose, gelatin and pectin) [62] with other materials such as elastomers and adhesive coatings. They require additional layers of adhesive to stay in place. Hydrocolloid-based bandages can offer good fluid absorption, but can change into a gel-like substrate, with the outer layer acting as a barrier for external impurities [51]. In addition, their structure may change with the amount of fluid absorbed, hence affecting their functionality to some degree.

Hydrogel can also be used to fabricate bandages, but it is rarely used as a standalone bandage and is commonly impregnated into a secondary bandage such as gauze or foam. Hydrogel bandages are made of naturally derived polymers, such as alginate, chitosan, and collagen, and are biocompatible crosslinked polymer networks with high H_2_O content that help to maintain a moist environment by delivering water molecules to the wound. They are used mainly when a cool environment is needed, as it is possible to keep the wound at 5 °C. In addition, hydrogel-based bandages were found to be more efficient than hydrocolloids [63] and gauzes [64], hence they need to be replaced less frequently. On the other hand, hydrogel bandages may suffer from stability issues as the result of pathogen transmission from the natural polymer source, and also from enzymatic degradation that may lower the matrix stability [65,66].

Transparent and nanofibrous films represent another type of bandage material, which is mostly made from self-adherent transparent polymer membranes (mostly polyurethane). They act as barriers to external impurities, ensure gas exchange and are atraumatic upon removal. As these bandages do not possess absorption nor swelling capabilities, they are highly elastic, transparent and very conformable to skin and other substrates. There are a variety of ways of manufacturing these films, such as electrospinning [67] and cutting and placing with outsourced converters; these may improve the final scalable manufacturing process.

In addition to the described bandages, there are additional types of wound dressings that are characterized as supplementary bandages for specific applications. For example, Tulle is a non-adherent dressing impregnated with paraffin for healing aid, and requires a secondary dressing to hold it in place [68,69]. Another example is a paper adhesive tape, which is used for approximating wound edges and is ideal for small wounds [70].

**Figure 5:**
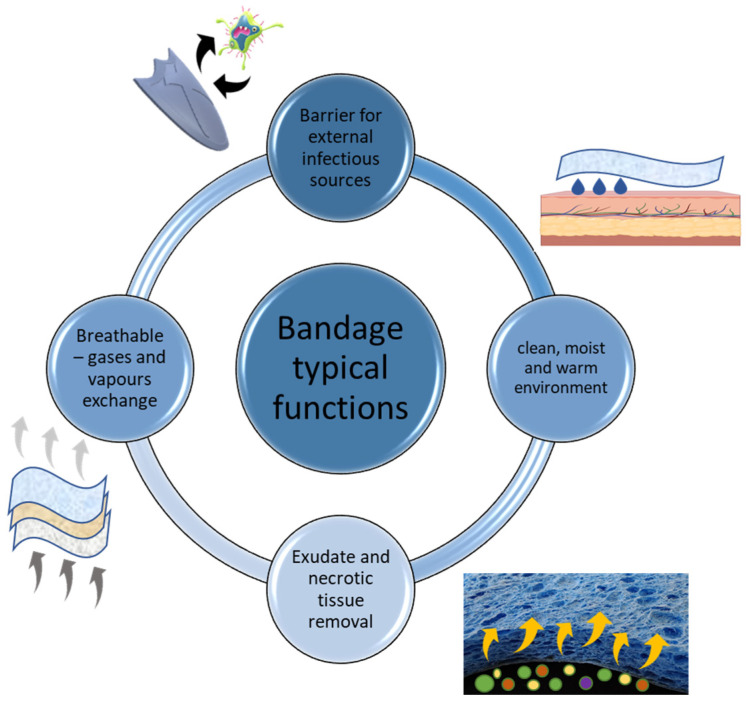
The main functions of the typical bandage for the improved wound-healing process.

With regard to the optimal wound treatments, an ideal bandage should possess the following characteristics: (1) bio- and surface-compatible, (2) good control of moisture, (3) air-permeable, (4) rapid removal of exudate and impurities, (5) protective against external impurities, (6) facile removal without discomfort, and (7) economical [12,68,71,72]. Additionally, the four main features that are necessary for improved wound healing are presented in Figure 5. These traditional bandages are ideal as a base for biosensors for medical, clinical and diagnostics applications for multiple reasons; these range from surface compatibility and adherence to the skin for mechanical biosensors and flexible electronics to the thermal management, high absorbency and gas exchange for electrochemical biosensors [73,74]. Table 1 summarizes the advantages and disadvantages of the aforementioned six bandage types, and shows that the desired characteristics of the ideal bandage are present in one or a combination of the following types of bandages.

## 4. Functional Bandages

**Figure 6:**
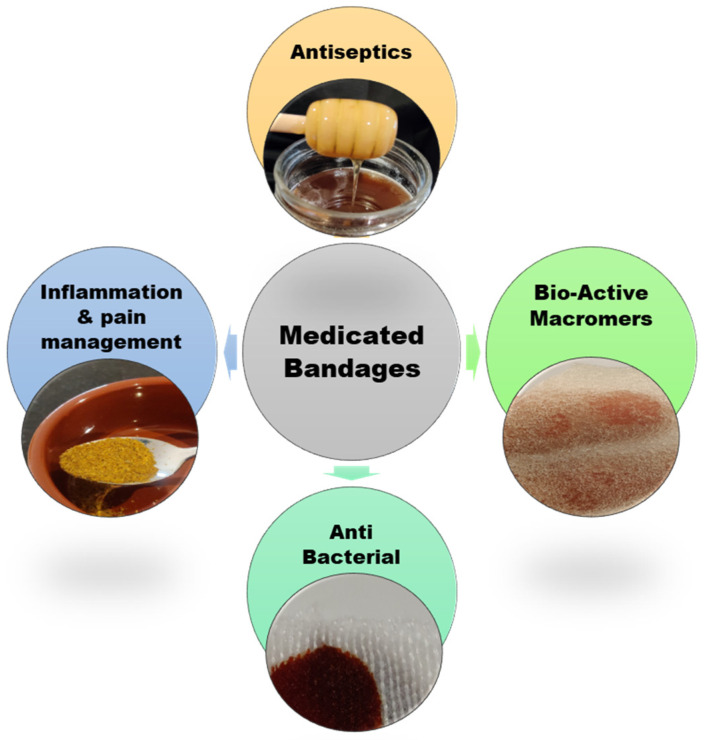
Four main types of medicated bandages: 1. Bio-Active Macromers: Actively enhance tissue regeneration by using natural and synthetic materials, (alginate, collagen, chitosan and PU-based polymers) [84,85], 2. Antiseptic: natural agents such as manuka honey and charcoal, and synthetics such as polyhexanide (PHMB) [86] 3. Anti-Bacterial agents such as silver, iodine and antibiotics. 4. Inflammation and pain management: drugs such as ibuprofen, lidocaine and more [87].

The next step in the evolution of the bandage as a healing accessory was to use previous knowledge and combine it with functional properties that take an active role in the healing process. These medicated bandages (Figure 6) are generally classified into two different groups based on the functionality of the active ingredient. The first group are designed for in situ drug release, such as anti-inflammatory, pain relief and anti-bacterial solutions, and can be described as a treatment for symptoms and external impurities [75]. The second group of bandages are classified as bio-active bandages that assist with endogenous activity by actively enhancing tissue regeneration and the body’s healing processes [76,77,78,79]. These bandages are an early example of drug delivery mechanisms that as a result of the immense amount of research in the last two decades, will become electronically controlled via surrounding stimuli, such as light, temperature or skin chemistry [80,81], or via directly received digital input [82,83].

### 4.1. Next-Generation Smart Wearable Bandages

The wearable market has grown significantly in recent years, and revolves mainly around the private consumer sector; in 2022, it reached a value of USD 61.4 billion [88]. The majority of wearables available today can be classified in four main groups: head-mounted, body-worn, lower body devices and wrist-worn or hand-held devices [1]. They can be described as mainly fashion items with limited sensory capability, where smartwatches, wristbands, rings, belts, and headbands are examples. Most current-generation devices have the same problem of being rigid and planar in comparison to human skin, which is soft and curvilinear [89]. These available wearable devices are mainly used for measurements of simple parameters such as heart rate [90]. Their accuracy has improved dramatically in the last couple of years, and the latest version of the Apple Watch offers electrocardiogram (ECG) recording capability that has been approved by the US Federal Drug Administration (FDA). Even so, the most advanced products available on the market still fail to continuously monitor basic vital signs during daily activity [91], mostly as a result of motion artifacts that originate from loose attachment to the skin [92]. These limitations remain the fundamental constraints on their measurement capacity, and hinder their accuracy and introduction into the healthcare system.

Wearable sensors started to appear in the second half of the 20th century. However, only in the last two decades, with the broad penetration of the Internet of Things (IoT), has their focus continuously evolved from simplifying and improving our everyday tasks to communication, data transfer, biofeedback and other sensory physiological functions [1,93,94]. With the continuous miniaturization of integrated circuits via the scaling of Moore’s law [95], and advancements in technology and material sciences, the available wearable devices have successfully become an integral part of the mainstream. However, with all the advancements in the field, the current generation of wearables still suffers from several issues, and they are far from being widely used in the healthcare system [96]. One roadblock is the interface between the sensor and the epidermis, where low conformity is a major factor of poor comfortability and poor accuracy. To improve the quality and reliability of the signals from the wearer to the sensor, some parameters need to be considered, such as adhesion to the skin for a more reliable contact, and the thickness of the adhesive layer to overcome the roughness of the skin’s surface (Table 2). In addition, bio-compatibility and gas-permeability are extremely important for prolonged use.

**Figure 7:**
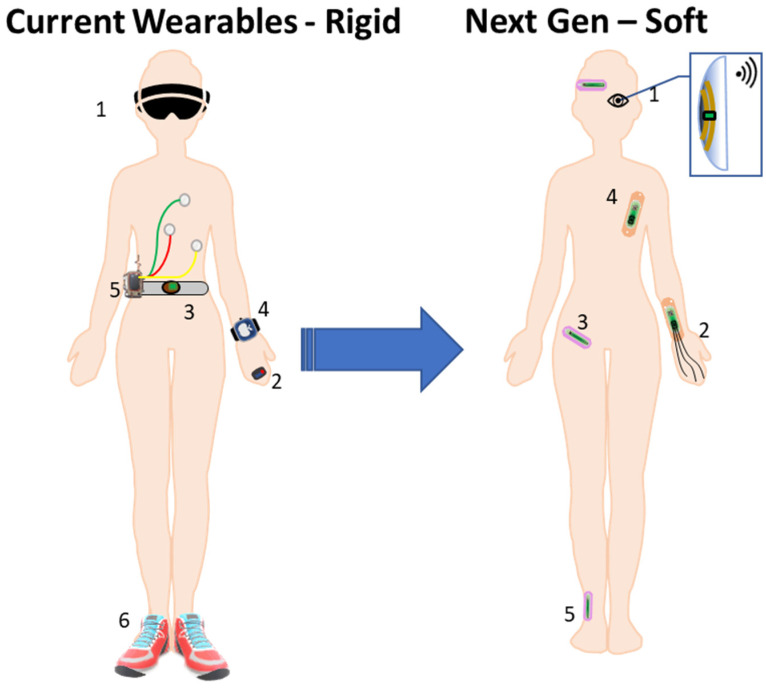
Bandage-based sensors of the current generation: 1. Smart glasses—augmented reality (AR) and communication (phone link). 2. Smart ring—payment method, wireless electronic mouse, oxygen saturation and more. 3. WELT Smart Belt Pro—fall prevention by sending a warning to the mobile if an abnormal gait pattern is detected.4. Smart watch—(blood pressure) BP, HR, activity, oxygen saturation, location and more. 5. Wearable ECG sensor. 6. Smart shoes—falling alert, posture correction, balancing and health monitor. Next generation. 1. Smart lens with ECU—AR and communication. 2. Strain sensor for control in the augmented and virtual reality (AR/VR) verses. 3. Strain sensors for body posture and movement detection. 4. Strain sensors and sweat sensors for vital sign monitoring. 5. Strain sensors for posture and posture monitoring.

In this context, the use of bandages as the base for wearables may help overcome the aforementioned issues to enable next-generation soft wearable biosensors. There are many stages for the sensor, or any wearable device for that matter, to overcome before it reaches the consumer for usage. From design to marketing, the two major hurdles for the final product to be approved and distributed are validation and acceptance by the user [2,102]. One potential solution to these structural disadvantages is to base the next generation of wearables on bandages. They are widely used in the medical field and are known for their versatility and ease of use [70]. This addresses the user’s acceptance of the product, as it is based on a commonly used item both in the healthcare system and privately worldwide. An additional merit of using smart bandages to replace the bulky and rigid devices with soft and commonly used substitutes (Figure 7) is that it will add a functionality factor (for example, a bandage that is capable of HR monitoring but that also has a drug delivery component and physical electrical stimuli [103]) and thereby might shorten the period of acceptance. The solution for the validation of the product is a more complex matter, as there are no standardized evaluations [104]. For it to be introduced into the healthcare system, a sensor must undergo a series of tests against laboratory-grade devices; however, there is no specific protocol for this comparison [105,106]. These laboratory-grade devices use straps and clinical grade measurement tools that diminish or even completely cancel the issues that arise from poor attachment to the skin. As bandages are conformal to the skin, made out of breathable materials and sometimes self-adherent, their use for sensory purposes could eventually diminish, if not annul, the gap between current medical-grade systems and available wearable sensors.

Recent advancements in the fields of material science [107,108,109], chemical diagnostic techniques [110,111], sensory design [112,113] and fabrication processes [114,115] present us with an immense data source in the bio-sensing field [116]. These advancements can be exploited to create a methodology for adapting the right technique and design to the bandage that can offer the best integration and data transfer from the body to the sensor. By knowing the type of sensor is to be used and the range of parameters to be surveilled, the correct set of properties from each layer (Figure 3) can be optimized. The bandages available on the market today come in different arrangements of structural layers, thicknesses, types of adhesive and sizes, and these structural distinctions can offer advantages for the desired specific sensor. For example, a transparent thin film bandage may be an excellent platform for a strain sensor; however, a bandage with a thicker layer of adhesive would be more suitable for a sensor tracking limb movements than for tracking heart rate. By using the range of properties available, such as elasticity, stretchability and thickness, [116] (Table 2) we can choose the desired attributes from different adhesives, substrates and available products, and match them to the designed sensor.

### 4.2. Categorization of Smart Wearable Bandages

The latest advancements in the field of biosensing can be divided into groups that differ by the sensory objective, such as biophysical and biochemical inputs [116], or by the functionality of the system, such as passive and active sensors [75]. The preceding differentiation is based on the properties that the sensor is designed to detect; for example, biophysical sensors measure the physical properties of a substance, such as a temperature, pressure, and mechanical stress [117]. These sensors typically use a physical mechanism to detect changes in the property they are measuring, and require a thin, adherent substrate to maximize the quality of the data acquired. On the other hand, biochemical sensors measure the chemical properties of a substance such as pH and glucose levels, or the presence of specific molecules. These sensors often use a biological component, such as enzymes or antibodies, to detect the presence of a specific chemical or molecule [118,119], and require a more absorbent medium between the sensor and the location at which it is applied. Both types of sensors, physical and chemical, are passive sensors that collect information from the epidermis and transfer it to a personal device, cloud, or directly to the healthcare provider. The latter differentiation between passive and active sensors is more based on the intricacy of the sensor’s function, which includes both the data collection and the delivery components [120], and as a result, the requirements of the bandages it is based on are broader. For both types of sensors, there are basic requirements from the substrates they are based on in addition to the specific requirements from each type of sensor, both of which bandages can provide for. The unique advantages, such as breathability and biocompatibility, of these approved and market-available products over traditional substrates enable us to prolong the period for which the sensor can stay on the epidermis. An additional advantage is better adhesion to the skin, which diminishes the noise from motion artifacts.

## 5. Passive Smart Bandages

**Figure 8:**
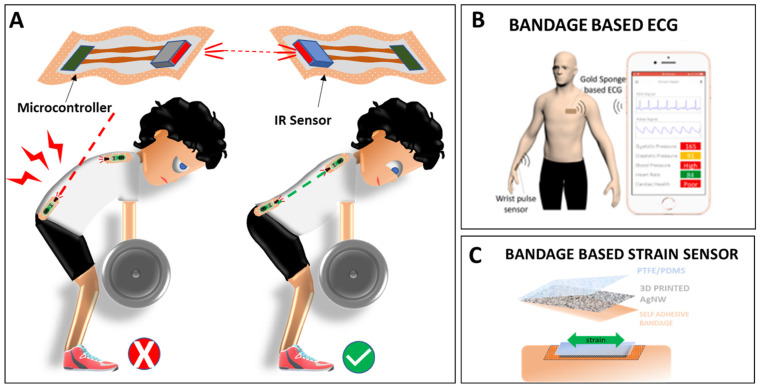
Examples for development of bandage-based and tattoo-based passive sensors for health monitoring. (**A**). Band-aid-based flexible wearable platform for fitness and athletics [134]. (**B**). Gold nanowire-integrated soft wearable system for dynamic continuous non-invasive cardiac monitoring. Reprinted with permission from Biosensors and Bioelectronics, Copyright 2022, Elsevier [128]. (**C**). 3D printed AgNW mesh as a resistive biophysical sensor.

With the goal of introducing wearable biosensors with real-time monitoring into mainstream broad use and the healthcare system in particular [121], the stream of information received by the sensor needs to be of uncompromising quality and reliability. In this context, several excellent reviews have already explored and elaborated on the features, materials and manufacturing processes researched [122,123]; however, the consistent issue is the nature of the interface between the epidermis and the sensor. The main purpose of a passive biosensor with focus on the healthcare system is to transfer data from the patient to the doctor, or any healthcare provider for that matter. As previously mentioned, the data that the sensors are designed to collect are biophysical and biochemical signals, for long periods, and as a result, the sensor’s base platform needs to be adapted to maximize the quality of the information received from the patient or product user. One example is thin film bandages serving as the basis for a kind of exercise monitoring, such as posture and movements (Figure 8A), for increased performance and greater effectiveness of the workout. In this case, higher endurance is needed from the bandage, and a thicker layer of both the adhesive and the film (layers A and B, Figure 3) should be considered. Additionally, biophysical targets such as temperature, body motion [124] and vascular and skin dynamics are signals that can be detected by electrodes based on low-modulus elastomeric materials (Figure 8B). These materials, such as PDMS [125] and SEBS, are combined with conductive fillers such as graphene [126], CNT’s and various conductive nanowires, such as AgNW [127] (Figure 8C), AuNW [128,129,130,131], or their combination [132,133]. In addition, advancements in optically active nanomaterials mean they present increased sensitivity to a variety of biomolecules [109]. Wearable sensors based on these methods need the extreme proximity to the surface that thin film bandages can deliver, in order to demonstrate increased sensitivity while carrying out their function as the base for the electrode and sensory array.

**Figure 9:**
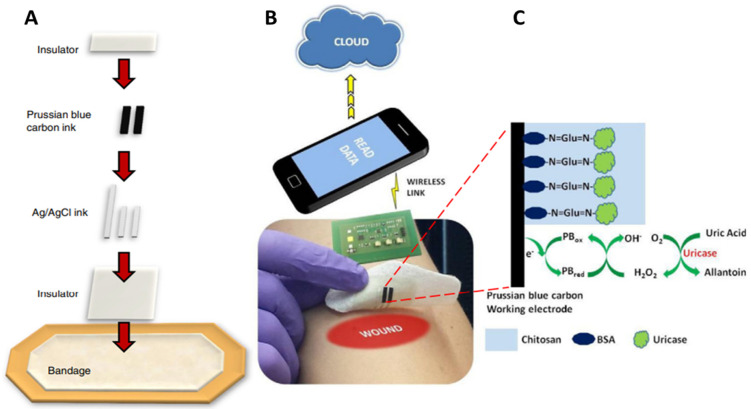
Biochemical sensors: (**A**). Screen printing the smart bandage. (**B**)**.** Wearable potentiostat determining uric acid concentration and wirelessly communicating with a computer or smartphone. (**C**). Schematics showing amperometric detection of uric acid with uricase immobilized on a PB working electrode. Reprinted with permission from Electrochemistry Communications, Copyright 2015, [119].

Another type of sensor is based on biochemical targets such as pH, ions, enzymes, DNA, and antibodies that provide additional information about the patient’s health status [135,136,137,138,139,140,141]. Although much progress has been made in the body fluid analysis methods, especially blood [142], traditional biochemical analysis still requires expensive instrumentation and trained personnel, in addition to being physically present in the hospital or laboratory, and an invasive sample collection process. Other body fluids such as saliva, tears and sweat may be considered noninvasive alternatives, and may help bypass the drawbacks of the conventional testing methods [143]. For a biochemical non-invasive sensor that acquires information from the epidermis layer, a more absorbent medium as the base for the sensor is required. Hydrogel and foam-based bandages are a great solution for these types of biosensors, as they deliver the body fluid from the surface directly to the electrode while remaining mostly unchanged [144] (Figure 9). Both solutions for biochemical sensors require a secondary layer with adhesive abilities, which increases the stability of the overall bandage structure, to present an adequate base for the electronic components.

When designing a wearable passive sensor, many parameters need to be taken in consideration for successful commercialization. With that said, the majority of the research available today focuses on the design, application and execution of the sensor itself, and certain topics are not weighted equally. Breathability and biocompatibility are an example of topics that are not being analyzed enough, even when they may be the reason a sensor will not progress beyond the designing bench. The lack of these attributes in the substrate that the sensor is fabricated on may result in the user experiencing discomfort or even an adverse reaction to the sensor. When adopting bandages in the initial stages of sensor design [145], these unwanted results can be avoided. Another topic that is being given more interest of late is the “comfort of wear” concept, wherein another merit of using bandages comes to the fore [146]. When examining the usability of passive sensors, both in the private market and healthcare system, an important approach is to take into consideration the level of disturbance to the wearer. By using a bandage that includes all desired parameters, such as flexibility, thickness and adhesion, and one that is already approved for commercial use, the sensor fabricated will be noticed as little as possible, and will therefore be easily accepted by the user. Another point of consideration in the development process is the fabrication method of the sensors. With the increasing demand for the fabrication of more complex and advanced sensors, the progress in this field is remarkable. The different methods with which to apply the sensor to the bandage vary from directly 3D printing the nanowire mesh onto the surface [147] to dip coating, transfer methodologies and laser printing [148], to name a few.

## 6. Active Smart Bandages

While the currently available technology in the wearables market is mainly based on data collection and monitoring, an additional kind of active device is slowly taking its place in the natural evolution of wearables. Active drug delivery systems have been on the market for several decades in items such as insulin pumps and do-it-yourself (DIY) blood analysis devices, but the technology available is still invasive, and sometimes requires a trained professional for initial setup [149,150]. With the current advancements in passive wearable sensors, transdermal drug delivery [151,152,153], flexible electronics, novel materials such as smart nanomaterials [111,154], and monitoring techniques [155,156], the addition of an active and externally controlled system is the obvious next step. The current active smart biosensors in development vary by many parameters; one example of these is bandages executing design objectives via reaction to external stimuli such as light, surface temperature and pH. Another approach is the direct execution of a set of actions sent directly from a control system such as the user’s portable phone [157] or the doctor’s healthcare management system [83].

**Figure 10:**
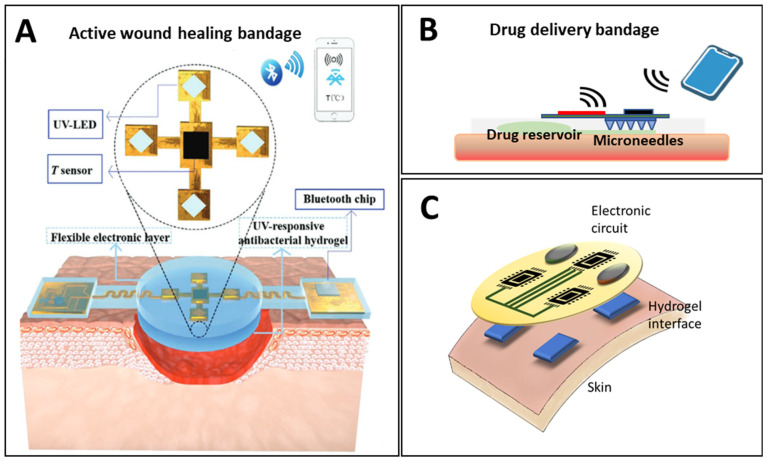
Responsive smart bandages with drug delivery capability by external and internal stimuli, (**A**). Externally triggered wound dressing: the on-demand release of therapeutic agents is activated through UV irradiation of a UV-responsive anti-bacterial hydrogel. Reproduced under the CC-BY license [83]. (**B**). Bandages-based drug delivery system layout. (**C**). Smart bandage with integrated biochemical sensors, stimulators and hydrogel interface for advanced wound care and accelerated healing.

When differentiating between the active sensors in development, the requirements of the bandages change according to their main design and structural purpose. Many active biosensors in research phases are centered around drug delivery mechanisms, which therefore dictates the characteristics needed from the interface between the epidermis and the biosensor [158]. Concentration gradient and other passive controlled therapeutic delivery mechanisms may not be suitable in many cases, and an external control unit is required for on-demand delivery. One example is the treatment of chronic wounds [159], in which the sensor’s objectives vary from controlling the wound environment through factors such as moisture and temperature to the introduction of drugs that enhance the healing process (Figure 10A). For these requirements, a hydrogel bandage would fit the criteria of the interface needed [160,161], as they enhance the compatibility with the skin through improved wetting ability which minimizes the air pockets between the device and the patient’s skin. Moreover, the control of the drug delivery is less energy demanding for both the amount of drug needed and the lower resistance of the skin, or the lack of it. Another example for which active sensors are more adequate is the treatment of a patient with severe or prolonged medical issues. The increased volume of drugs needed and the real-time continuous monitoring in these cases require more energy-dense and electronically complex systems, and the bandage substrate’s purpose adds increased structural requirements. Microneedles and drug reservoirs (Figure 10B) are an example of an increased drug delivery system that may be used in these cases, in which a more adherent substrate is needed for firm positioning of the delivery mechanism. These advanced and complex sensors require an advanced architecture of bandages that can be achieved by combining several existing products into one multi-purpose platform. An electrical stimulus is another method for the enhancement [103] of chronic wound treatment, and is an additional point to be considered. As advancements in the field are remarkable, self-powered bandages [162] and wearable self-sustained diagnostic systems are not far off in the future. With that said, the same attributes that the passive sensors require for adaptation are still needed for active sensors, especially if the final product is more cumbersome. The comfort of wear, product adoption and validation points must be considered to a similar extent if not more than in passive sensors, as these advanced sensors are bigger, more complex and have a direct response to the user’s health condition. Using products that have an existing foothold in the current consumer market, the way to market may be shorter and more economically viable.

## 7. Bandage Manufacturing

Any new product to be introduced into a developed and growing market needs to be designed with the objectives of scale-up and mass production at the top of the list. There are many risk factors to take in consideration, such as partnerships for specific production stages that might not come to fruition [163], or a raw material of choice that might be discontinued. From a more global point of view, logistical reasons, such as the cost of shipping [164] and legislation differences between countries, may be some of the reasons that the product does not leave the research board and advance to the next stage. By using an existing validated and approved product, these risks can be mitigated. In addition, designing the desired sensor with a globally available product from an international conglomerate may result in a faster, leaner and more economical path to the market. An additional advantage of using an existing product that is globally used and accepted both in the consumer and the healthcare markets is the available designated waste management system. The existing disposal methods for healthcare waste have gone through many changes in response to the pandemic, and continuous improvements have been introduced to them. This may have a meaningful impact on the disposal of the next generation of wearables when compared to the current increasing environmental burden and disposal issues of e-waste from current-generation wearables.

**Figure 11:**
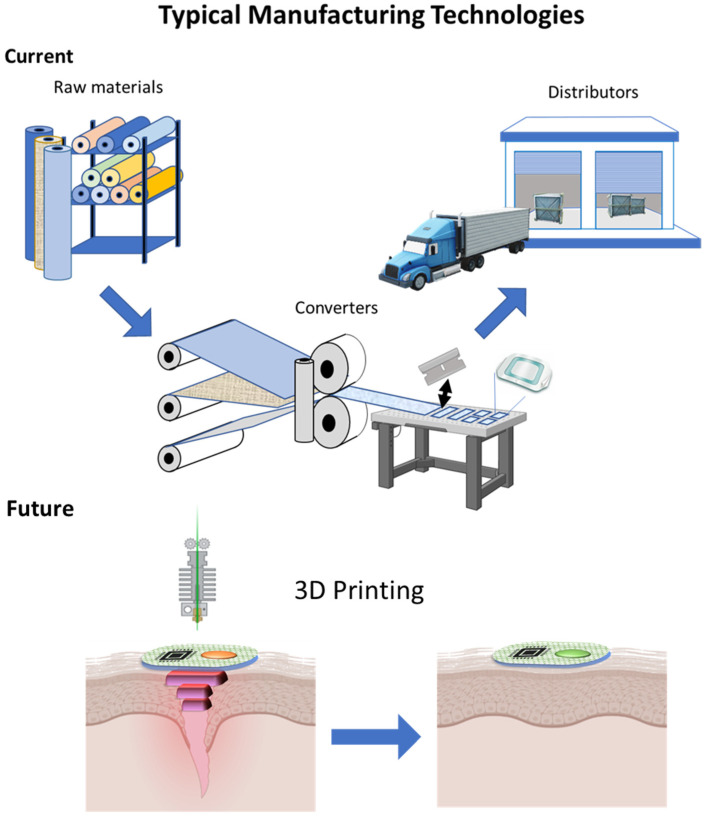
Three main stages in the typical industrial process. Manufacturers of raw material: adhesive layers, backing and barrier layers. Converters: combining the layers of raw materials, cutting by product size (manufacturing plans), sensor and electronics integration and packaging by order. Distributors: delivery directly to healthcare providers or pharmacies. 3D Printing: advancements in the manufacturing process of bandages and future use of 3D printing technology for direct deposition of both the bandage and electronics on skin.

The use of existing products can be divided into two main approaches. The first is the use the raw materials (Figure 11, Current) which are manufactured by the companies as part of their bandage assembly process. The main advantage of using existing materials from leading manufacturers such as 3M, Smith & Nephew and Johnson & Johnson is avoiding the aforementioned hurdles of availability and global approval. The direct result is that the intense testing and long waiting periods for certification are made redundant. In addition, the use of the raw materials directly in layers B, C and D (Figure 3), and not the final product that is available on the market, may be adapted to simplify the testing stages of the research and the creation of the prototype.

The second approach to using existing products is more oriented towards the scale-up stage, or closer to the final mass production stage. Converters are external companies that specialize in further processing, modifying or combining the manufacturer’s raw materials into the desired and new final product. Most international companies have the capability of in-house manufacturing, although it is designated for the bulk of their final products that eventually reach the consumer market. With that said, the services of converters are a necessity in the ever-evolving global market. For geographical reasons, it is more economical to ship the raw materials in bulk to a country with no production plants, and assemble the final product locally using a certified converter. Moreover, the manufacturing of low production volume products is more economically feasible with this course of action. In the case in which a small private company, start-up or even a research team wishes to assemble a prototype using specific materials from a selected company, it is a matter of reaching out to the closest converter [165].

The emerging new approaches for future bandage manufacturing are 3D printing (Figure 11 Future) and electrospinning directly onto the consumer’s skin. Recent technical developments in the field of 3D printing have brought the idea of printing a smart bandage directly onto the patient’s skin. As 3D printing is based on a computerized process, it is highly reliable, cost-effective and can be used to implement a complex and multi-material design [166]. In addition, with this approach, all stages and components can be applied as part of the same process. Moreover, 3D-printed hydrogels have been considered for medical purposes [167,168], including wound healing and drug delivery. Another approach is the electrospinning [163,169,170,171] of nanofibrous dressings, but this approach is less versatile and can be part of a manufacturing process; however, unlike the 3D printing, it cannot cover all the stages necessary for the manufacturing of the final product.

## 8. Conclusions and Future Perspectives

With all the advancements in the wearable bio-sensors field, the use of existing products for the introduction of a fully autonomous device that serves as a portable healthcare professional will have an enormous impact on society. With that said, some challenges still prevent compatible and skin-like devices reaching health care systems around the world: a uniform standard for the validation of wearables, the acceptance of the product by the end-user and a mass production process with an acceptable cost-to-benefit ratio, to name a few. When looking into the five millennia-long journeys of the evolution of bandages and the field of wound treatments, the similarities to wearables are striking, and may offer some acceptable solutions for the challenges mentioned. Most films, foams, hydrogels and composite bandages available today undergo the same cumbersome validation process that future wearables will eventually have to surpass, and simultaneously offer desirable features for the design of wearable biosensors. This article critically reviews bandage-based wearable biosensors by covering the discussion of the historical development of bandages, their material composition, design and manufacturing, as well as their attributes in user acceptance for anytime, anywhere health monitoring in the future of The Medical Internet of Things. When considering the use of bandages as the base of future wearables, we can harness the knowledge that was accumulated during this long voyage, both in the manufacturing and the delivery of a product to the consumer. By doing so, we might expedite the development of future healthcare systems simply by advancing smart wearable bandages.

**Figure 12:**
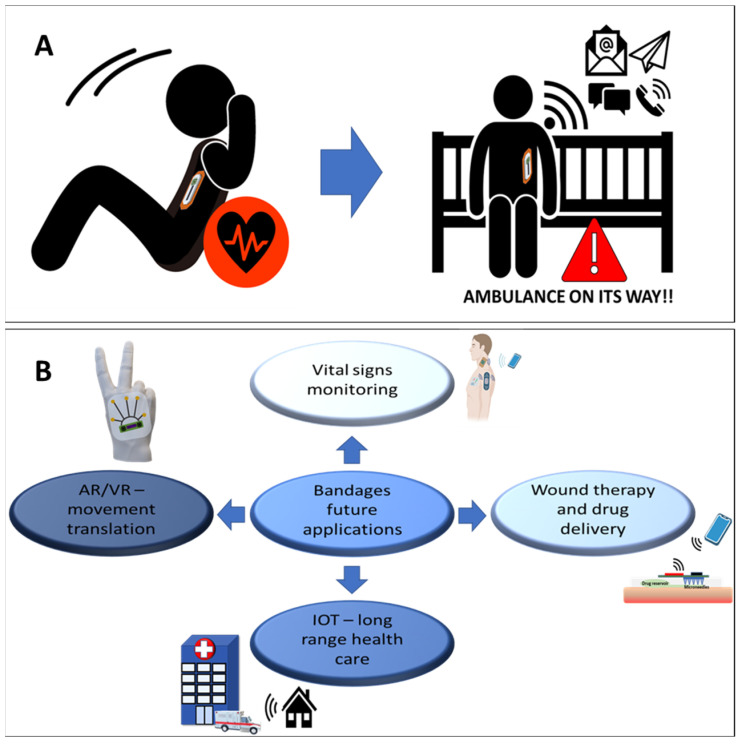
(**A**). Future uses of bandage-based biosensors; the smart bandage detects a physiological disorder and directly contacts the healthcare provider. Exercising person silhouette downloaded from https://pixy.org/1104016/, accessed on 9 January 2023. (**B**). An overview of the future possible applications based on smart bandages for improved patient health analysis, and better support for medical staff requirements.

Developments in the fields of medicine, materials, electronics and artificial intelligence (AI) [172] will eventually bring the smart wearables industry to everyday life, and it will become an essential part of everyday life. Imagine waking up in the morning, sticking a bandage to your chest, going out to exercise, showering and going straight to the office, all while the bandage continuously monitors your well-being and fitness and even proactively intervenes in case of an emergency (Figure 12A). To expedite the process of acceptance and approval by administrative systems, the use of existing products and the harvesting of accumulated knowledge in the mass production and manufacturing practices of medical devices is extremely beneficial for the introduction of wearables into the mainstream. Healthcare product manufacturers such as 3M have a vast database and have accumulated great knowledge in the manufacturing of bandages and microfluidic devices [47], and even in consulting services to startups at the product-to-market stage [173,174]. When working on a new wearable sensor, the materials used in its design are mostly chosen based on their technical properties; attributes that are important for the longevity of the product, comfort and even validation processes are often overlooked. Latex, for example, is used in some forms for strain sensor development, but if industry standards are to be considered, it is classified as a material of concern. ISO 10993 [175], for example, is the standard in biocompatibility that evaluation studies are performed in accordance with, and it classifies it as such. Another point to be considered in the validation process is the accelerated aging tests carried out on devices to evaluate their performance after a long period of use. Nine weeks at 50 degrees Celsius is the equivalent of a year of use, and when choosing the adhesive or the material of use for a wearable device, this should be taken in consideration but is often overlooked.

When considering their versatility and attempting to forecast the future applications of wearables in the healthcare system (Figure 12), the importance of end-user acceptance and the approval process of governing bodies are often underestimated. Choosing bandages as the basis of wearables could resolve all these issues by introducing well-established and knowledgeable industry partners into the development process, in conjunction with multidisciplinary academic researchers.

## Figures and Tables

**Table 1:** Advantages and disadvantages of the main bandages on the market.

Bandage Type	Advantages	Disadvantages	Highlights for Manufacturing	References
Gauzes	Highly absorbent	Fibber and particulate loss	Excellent for holding sensors in place at any interface of the body.	[12,25,51,52]
	Surface compatibility	Bacteria contamination	The structure may change with fluid absorbed or evaporated from the bandage	
	Economical	Discomfort in dry-state removal	Widely used and very economical	
Hydro-conductive	No removal discomfort	High frequency of replacement	Excellent absorption for biochemical sensors	[51,52]
	Rapid removal of exudate and impurities		The frequency of replacement makes it an expensive option for mass production	
Foam	Highly absorbent	High frequency of replacement for infected wounds Tendency to swell and create air pockets between the wound bed and bandage	Excellent for both biophysical and biochemical sensors	[12,14,52]
	Usable for long periods if the surface is not infected		Little to no structural changes with absorption (depends on foam density),	
	Allows gas exchange	Wound maceration	Requires an adhesive layer to eliminate artefacts associated with motion	
	Thermal management through insulating			
Hydrogel	No removal discomfort	Not a good barrier for external impurities	Not very economical due to secondary dressings and manufacturing costs	[12,48,52,53,54,55]
	Allows gas exchange	High frequency of replacement for infected wounds	Excellent absorption with no extreme structural changes makes it an excellent base for biochemical sensors	
	Keeps a moist environment			
	Thermal management through cooling	Secondary dressing required		
Hydrocolloid	Highly absorbent	Not suitable for infected wounds	Excellent absorption but with extreme structural transformations as a result	[12,52,54]
	The barrier to external impurities	Discomfort and possible trauma during removal		
	Keeps a moist environment			
Transparent Film	No removal discomfort	Not suitable for infected wounds	Good structural consistency for biophysical sensors	[12,51,52]
	The barrier to external impurities		Excellent conformity to the epidermis layer	
	Keeps a moist environment	Not absorbent	Proven mass production process with a clear scale-up process	
	Easy application			
	Allows gas exchange			

**Table 2:** Mechanical properties of existing substrates used for wearable sensors and bandages in comparison to human skin.

Product	Chemical Composition	Elastic Modulus	Adhesive Force (N)	Thickness (μm)	Reference
Human skin	N/A	10–500 kPa	N/A	50–1500	[97]
TPE 45A	SEBS	0.02 Gpa	N/A	N/A	[98]
EcoFlex	Silicon elastomer	69 kPa	0.24	5–25	[99]
Spray-on-bandage (3M)	Acrylate terpolymer, Polyphenylmethylsiloxane, Hexamethyldisiloxane	85 MPa	0.98	1	[99]
Tegaderm film (3M)	Acrylate terpolymer, Polyurethane, Polyester, Silicone film	12 MPa	1.02	35	[99]
Silicon tape (3M)	Silicone adhesive, Acrylate polymer, Thermoplastic, polyester	127 MPa	1.37	330	[99]
PDMS	silicon elastomer	2 MPa	0.22	5–25	[97]
PET	Bis(2-hydroxyethyl) terephthalate	2.5 GPa	N/A	2.5–6	[100]
Acrylic adhesive	Methacrylic polymer	17 kPa	16	12–60	[101]

## Data Availability

Not applicable.
